# Tuberculosis and Melioidosis at Distinct Sites Occurring Simultaneously

**DOI:** 10.1155/2020/9818129

**Published:** 2020-01-03

**Authors:** Seow Yen Tan

**Affiliations:** Department of Infectious Diseases, Changi General Hospital, Singapore

## Abstract

**Rationale:**

Both tuberculosis and melioidosis are commonly encountered infectious diseases in South East Asia. However, these conditions occur commonly in isolation, cases of coinfection of *Mycobacterium tuberculosis* and *Burkholderia pseudomallei* are rare. These cases report of the isolation of both organisms concomitantly in a single disease site. We report the first case of concomitant infection at distinct noncontiguous sites.

**Patient Concerns:**

A 64-year-old man, with chronic alcohol consumption, presented with a month long history of left-sided abdominal pain, as well as significant weight loss and fever prior to the onset of abdominal pain.

**Diagnosis:**

Disseminated tuberculosis with pulmonary and gastrointestinal involvement and a splenic abscess due to melioidosis.

**Interventions:**

The patient was treated concomitantly for pulmonary and gastrointestinal tuberculosis, as well as a splenic abscess due to melioidosis.

**Outcomes:**

The patient is reported to be well, with resolution of symptoms, as well as radiological resolution of the splenic abscess.

**Lessons:**

Both melioidosis and tuberculosis can present with a similar clinical picture, and coinfections are rare. Hence, increased awareness among clinicians and microbiologists can help in diagnosing both diseases even when it is not clinically apparent.

## 1. Introduction

This case highlights a patient who presented with a subacute history of abdominal pain and subsequently found to have pulmonary as well as colonic tuberculosis. He was subsequently discovered to have melioidosis occurring concomitantly in the spleen.

Melioidosis is an infectious disease associated with high mortality and morbidity in endemic regions of South East Asia and northern Australia [1]. The etiological agent, *Burkholderia pseudomallei*, can be found in the soil and water in these endemic regions. Infection generally occurs through inhalation, ingestion, or contact of skin wounds with contaminated soil, dust particles, or water. Melioidosis, also known as the great mimicker, can result in highly diverse disease manifestations, often complicating diagnosis and delaying treatment. Tuberculosis is caused by *Mycobacterium tuberculosis*, and it primarily affects the lungs. Nonetheless, it can affect any organ. Though, the incidence of tuberculosis has decreased in Singapore (37.6 per 100,000 resident population in 2014), it remains a disease of public health importance worldwide [2].

Coinfections of tuberculosis and melioidosis have been reported in the past, but these cases describe the isolation of both organisms within the same site of infection. This case is unique that it describes a patient who suffers from both melioidosis and tuberculosis affecting multiple noncontiguous organs at the same time.

## 2. Case Presentation

The local Institutional Review Board affiliated to the author's institution determined that ethics approval was not required for this case report. Consent has been obtained from the patient for publication of the case report. All identifying information of the patient has been removed.

We report the case of a 64-year-old man, residing in Singapore, with a known history of hypertension, hyperlipidaemia, and gout, and he has never been on any long-term medications for all his medical conditions. He presented with one month of left-sided abdominal pain, and a weight loss of 15 kg over one year. He also experienced night sweats, chills, and rigors for approximately 3 months. He denied any cough or shortness of breath. He reported consuming two bottles of beer daily for the past 40 years and smoking 20 cigarettes daily for the same duration. He also complained of intractable hiccups for a week, which interrupted his sleep. He used to work as a cable jointer and was stationed at various parts of the world, including the African continent, and multiple countries in South East Asia.

On examination, the patient was febrile on admission with a temperature of 38.2°C, blood pressure was 103/66 mmHg, and a heart rate of 99 beats/min. He was alert, but lethargic, and mucous membranes were dry. Heart sounds were dual, and the lungs were clear on auscultation. His abdomen was soft, but tender at the left hypochondrium, with no guarding, or rebound tenderness. Digital rectal examination showed fresh blood stains, with no palpable rectal masses.

**Figure 1:**
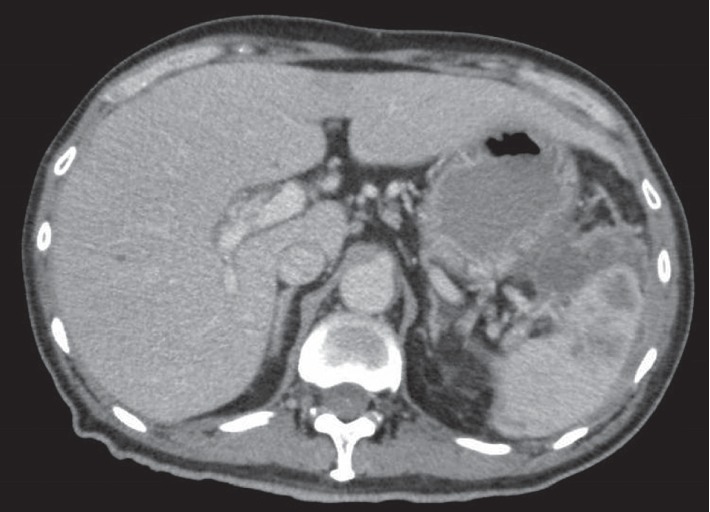
Computed tomography image of splenic abscess.

Initial laboratory investigations showed an elevated creatinine (147 *μ*mol/L, reference range 65–125 *μ*mol/L), hyponatremia (126 mmol/L, reference range 135–145 mmol/L), and borderline hyperkalaemia (5.2 mmol/L, reference range 3.5–5.3 mmol/L). The alkaline phosphatase was elevated at 444 U/L (reference range 32–103 U/L), with alanine transaminase (ALT) at 90 U/L (reference range 10–55 U/L). Albumin level was 45 g/L (reference range 37–51 g/L). Full blood count showed a hemoglobin level of 14.7 g/dL (reference range 13–17 g/dL), white blood cell count was 10.88 × 10^3^/*μ*L (reference range 4–10 × 10^3^/*μ*L), and platelet count was 296 × 10^3^/*μ*L (reference range 150–450 × 10^3^/*μ*L). His HIV screen was nonreactive. Chest radiograph showed an area of parenchymal scarring in the right upper zone and bilateral apical pleural thickening. Abdominal X-ray was unremarkable. A computed tomography (CT) of the chest, abdomen, and pelvis showed small hypodensities suggestive of multiple microabscesses of the spleen, with an irregular loculated rim-enhancing extracapsular collection adjacent to the splenic flexure, measuring 5.2 cm × 3.5 cm (see Figure 1). The CT scan also revealed a mass-like consolidation in the apical segment of the right upper lobe with adjacent tree-in-bud centrilobular nodularity.

Scanty amount of acid-fast bacilli (AFB) were seen on Ziehl–Neelsen staining of the sputum, and the Tuberculosis GeneXpert™ was positive as well. The patient was started on the standard first-line regimen of tuberculosis with rifampicin, isoniazid, ethambutol, and pyrazinamide. AFB were subsequently seen in the stool samples as well. The AFB cultures from both sputum and stool eventually yielded a pansusceptible *Mycobacterium tuberculosis*. He was discharged shortly after antituberculous therapy was initiated.

Two weeks later, the patient was readmitted after he developed severe lethargy and left big toe pain. He also had intractable hiccups that have not resolved since his previous admission. Clinical examination revealed mild right hypochondrial tenderness, erythema, and tenderness at the left first metatarsal phalangeal joint. Laboratory investigations showed elevated aspartate transaminase (AST) and alanine transaminase (AST more than ALT) and serum uric acid. Pyrazinamide was discontinued.

The patient's symptoms improved after stopping pyrazinamide, with the exception of the hiccups. Decision was made to perform a percutaneous drainage of the perisplenic collection which yielded pus and resulted in resolution of the hiccups. The fluid culture eventually grew *Burkholderia pseudomallei*. Tuberculosis Probetec™ and AFB culture were negative.

He was continued on the standard first-line regimen of tuberculosis, with a step down to rifampicin and isoniazid after two months of induction treatment. The patient was reportedly compliant on self-administered therapy. He was started on six weeks of intravenous (IV) ceftazidime for the treatment of melioidosis, in the Outpatient Parenteral Antibiotic Therapy (OPAT) centre. Unfortunately, the patient had fever as well as agranulocytosis on Day 40 of IV ceftazidime. This resulted in yet another hospital admission.  Table 1 shows a summary of the medications used in the treatment of tuberculosis and melioidosis.

A repeat CT scan showed resolution of the perisplenic abscess, there was improvement in the lung infiltrates, and there were no new intraabdominal collections. His fever and neutropenia subsequently resolved 2 weeks later, after discontinuing IV ceftazidime, and given pegfilgrastim. The induction phase of the treatment for melioidosis was completed inpatient using IV meropenem and was then switched to oral cotrimoxazole for further treatment of melioidosis. However, the patient defaulted subsequent follow-ups and did not comply with treatment for melioidosis. He had not been admitted to any hospital since he defaulted follow-up appointments for his melioidosis. Hence, it was presumed that the melioidosis had not relapsed.

In view of noncompliance to self-administered therapy for tuberculosis, he was referred to the National Tuberculosis Control Unit (TBCU). He completed six months of antituberculous therapy via directly observed therapy (DOT) under the supervision of TBCU. He was reported to be well during the follow-up visits for DOT and subsequently discharged from TBCU follow-up.

## 3. Discussion

This case is the first reported case of tuberculosis and melioidosis at distinct sites, occurring simultaneously. Cases of coinfection have been reported in Asia; however, these occurred within the same anatomical site [3–7].  Table 2 summarises the clinical features and profile of the reported cases. These cases occurred not only within the known endemic areas in South East Asia, but also where melioidosis is emerging, in India, with imported cases reported in South Korea.

Hazardous alcohol use was the only risk factor that was identified in our patient. The other important known risk factors such as diabetes, chronic liver disease, and chronic lung disease were notably absent [8]. His retroviral screen also returned negative. He probably had a latent infection with melioidosis, acquiring it during his duties as a cable jointer abroad few decades ago, and it reactivated eventually, together with tuberculosis. The symptomatic disease was probably triggered by a waning immune system, associated with aging and long-term alcohol consumption.

Nevertheless, it is also likely that he acquired the infection locally in Singapore. The overall annual incidence rate of melioidosis in Singapore was reported to be at 1.1 per 100,000 population. Diabetes mellitus is a key comorbidity in this study [9]. The patient's occupation also put him at risk of being infected with melioidosis. High-risk occupations include trucking/machine operators and those involved in the farming and agricultural industry due to regular soil exposure [10]. The patient's job as a cable jointer involved making and repairing joins in insulated power supply and control cables installed in underground pipes and trenches.

The diagnosis of melioidosis would have been missed if the patient did not undergo percutaneous aspiration of the perisplenic collection, done only to relieve the intractable hiccups. Upon the initiation of tuberculosis treatment, there was resolution of fever and the patient had felt better; hence, there were no plans to drain the splenic abscess initially. Disseminated tuberculosis could adequately explain all the clinical manifestations the patient had. However, the intractable hiccups, as a result of diaphragmatic irritation from the perisplenic abscess, were slow to respond to antituberculous therapy. This could have been an indication that there was a second pathology although the slow clinical response could be attributed to lack of source control as no drainage of the perisplenic abscess was done. A study in Singapore has indicated that the most common etiological agent encountered in splenic abscesses was *Burkholderia pseudomallei*, and tuberculosis was not identified as a cause of splenic abscess in this study [11].

In a patient who has an apparent unifying diagnosis, it is still important to consider a second pathology especially if the patient's progress is not consistent with the treatment rendered. If the perisplenic abscess had been due to tuberculosis, we could expect the hiccups to reduce in frequency and intensity. The original intention of the percutaneous drainage was for symptomatic relief, but we ended up elucidating a second pathology. Both tuberculosis and melioidosis can result in disseminated infections, and they are similar as clinical manifestations vary widely. There are also no distinguishing clinical and radiological features that can reliably differentiate the two conditions, making the diagnostic process even more challenging. Hence, it is important to maintain a reasonable index of suspicion, when managing patients in an area where coinfections have been increasingly reported. This is important to ensure that patients are given optimal care and treatment, despite the complexity of their condition.

## 4. Conclusion

This case illustrates that concomitant tuberculosis and melioidosis infection can occur not only in contiguous sites. Treating physicians should keep an open mind when encountering such a patient and be mindful of the local disease epidemiology.

## Figures and Tables

**Table 1:** Summary of pertinent laboratory investigation results and medications used for treatment of tuberculosis and melioidosis.

Baseline results	Initiation of tuberculosis treatment	2 weeks into tuberculosis treatment	Initiation of treatment of melioidosis	6 weeks into treatment of melioidosis
ALT 90 U/L	Rifampicin 600 mg OD	ALT 253 U/L	Rifampicin 600 mg OD	ALT 31 U/L
AST 46 U/L	Isoniazid isoniazid 300 mg OD	AST 526 U/L	Isoniazid isoniazid 300 mg OD	AST 91 U/L
Bil 11 *μ*mol/L	Ethambutol 900 mg OD	Bil 90.7 *μ*mol/L	Ethambutol 900 mg OD	Bil 15.7 *μ*mol/L
ALP 444 U/L	Pyrazinamide 1.5 g OD	ALP 444 U/L	Ceftazidime 2 g Q8H	ALP 56 U/L
Creat 147 *μ*mol/L		Creat 99 *μ*mol/L		Creat 157 *μ*mol/L
Hb 14.4 g/dl		Hb 14.2 g/dl		Hb 10.8 g/dl
WBC 10.88 × 10^9^/L		WBC 8.0 × 10^9^/L		WBC 1.1 × 10^9^/L
Plt 298 × 10^9^/L		Plt 206 × 10^9^/L		Plt 217 × 10^9^/L

Hemoglobin (Hb) 13–17 g/dL, white blood cell count (WBC) 4–10 × 10^3^/*μ*L, platelet (Plt) 150–450 × 10^3^/*μ*L, creatinine (Creat) 65–125 *μ*mol/L, alanine transaminase (ALT) 10–55 U/L, aspartate transaminase (AST) 10–45 U/L, bilirubin (Bil) 5.0–30.0 *μ*mol/L, albumin (Alb) 37–51 g/dL, alkaline phosphatase (ALP) 32–103 U/L, and once daily (OD).

**Table 2:** Clinical features and demographics of patients with reported melioidosis and tuberculosis coinfection.

	Our case	Sulaiman et al. [3]	Azali et al. [4]	Shetty et al. [5]	Shenoy et al. [6]	Kim et al. [7]
Country	Singapore	Malaysia	Malaysia	India	India	Korea
Age/gender	64/male	54/male	49/male	40/male	40/male	60/male
Risk factor	Chronic alcoholism	Diabetes mellitus	None	Diabetes mellitus	Diabetes mellitus	None
Occupational exposure	Cable jointer	Palm oil plantation worker	Agricultural department staff	Agriculturist in paddy fields	Paddy field worker	Welder
Sites of infection	Gastrointestinal tract, lung, splenic abscess	Cervical abscess	Liver abscess	Lung	Neck abscess	Pneumonia
